# Dynamic recruitment of resting state sub-networks

**DOI:** 10.1016/j.neuroimage.2015.04.030

**Published:** 2015-07-15

**Authors:** George C. O'Neill, Markus Bauer, Mark W. Woolrich, Peter G. Morris, Gareth R. Barnes, Matthew J. Brookes

**Affiliations:** a Sir Peter Mansfield Imaging Centre, School of Physics and Astronomy, University of Nottingham, University Park, Nottingham NG7 2RD, United Kingdom; b School of Psychology, University of Nottingham, University Park, Nottingham NG7 2RD, United Kingdom; c Oxford Centre for Human Brain Activity, University of Oxford, Warneford Hospital, Oxford OX3 7JX, United Kingdom; d fMRIB Centre, University of Oxford, John Radcliffe Hospital, Oxford OX3 9DU, United Kingdom; e Wellcome Trust Centre for Neuroimaging, University College London, 12 Queen Square, London WC1N 3BG, United Kingdom

## Abstract

Resting state networks (RSNs) are of fundamental importance in human systems neuroscience with evidence suggesting that they are integral to healthy brain function and perturbed in pathology. Despite rapid progress in this area, the temporal dynamics governing the functional connectivities that underlie RSN structure remain poorly understood. Here, we present a framework to help further our understanding of RSN dynamics. We describe a methodology which exploits the direct nature and high temporal resolution of magnetoencephalography (MEG). This technique, which builds on previous work, extends from solving fundamental confounds in MEG (source leakage) to multivariate modelling of transient connectivity. The resulting processing pipeline facilitates direct (electrophysiological) measurement of dynamic functional networks. Our results show that, when functional connectivity is assessed in small time windows, the canonical sensorimotor network can be decomposed into a number of transiently synchronising sub-networks, recruitment of which depends on current mental state. These rapidly changing sub-networks are spatially focal with, for example, bilateral primary sensory and motor areas resolved into two separate sub-networks. The likely interpretation is that the larger canonical sensorimotor network most often seen in neuroimaging studies reflects only a temporal aggregate of these transient sub-networks. Our approach opens new frontiers to study RSN dynamics, showing that MEG is capable of revealing the spatial, temporal and spectral signature of the human connectome in health and disease.

## Highlights

• The sensorimotor network consists of a series of transiently synchronising subnetworks.
• These subnetworks are robust across multiple tasks.
• The occurrence of these subnetworks is modulated by the current mental state.

## Introduction

Recent years have seen a new frontier in human neuroimaging brought about by the measurement of functional connectivity between brain regions. The finding of statistical interdependencies between signals representing brain function in spatially separate areas, even in the absence of a task (so called “resting state” connectivity) was shown by Biswal et al. (1995) and has subsequently been confirmed in many papers (e.g. Beckmann et al., 2005; Fox et al., 2005; Raichle et al., 2001). The principal finding is that brain function is supported by a relatively small set of large scale distributed networks, each characterised by spatially resolved patterns of functional connectivity. Some networks support sensory function (e.g. the sensorimotor network), whilst others are associated with attention and cognition (e.g. the fronto-parietal networks). These networks are reproducible across subjects, present in resting and task positive data, and are perturbed in a variety of pathologies. The mathematical methods most commonly used to probe functional connectivity employ a measurement of temporal correlation calculated over large time windows, usually comprising the entire experiment. This necessarily implies a prior assumption that functional connectivity between regions is stationary. However, increasing evidence from recent studies (Allen et al., 2014; Baker et al., 2012, 2014; Brookes et al., 2014; Chang and Glover, 2010; de Pasquale et al., 2010; Hutchison et al., 2013) suggests that resting state networks (RSNs), and the functional connectivities that define them, are time dependent. These dynamics are poorly understood, but the likelihood is that healthy brain function is supported by rapid and transient formation and dissolution of many small focal networks, the dynamics of which depend on current processing load. Furthermore, the RSN signatures that are commonly depicted in neuroimaging studies (henceforth termed ‘static’ RSNs) doubtless represent a time average of this transient connectivity. In this paper, we present a new framework in which to investigate and understand RSNs, by showing explicitly that multiple transiently synchronising sub-networks underlie the static network topology of the sensorimotor system.

Functional magnetic resonance imaging (fMRI) remains the most common means to investigate RSNs, and recent fMRI studies provide evidence for network non-stationarity (see (Hutchison et al., 2013) for a review). Indeed transient connectivity measures elucidate significant departures from established RSNs (Allen et al., 2014), with networks observed to form and dissolve over time. This said, a limitation of fMRI is the slow and indirect haemodynamic response, which makes measurement of fast temporal dynamics difficult. Recent years have seen a rapid advance in our understanding of neural oscillations (rhythmic *electrical* activity within cell assemblies). These oscillations, commonly reported in the 1 Hz–200 Hz band, are thought to represent an intrinsic mode of electrophysiological connectivity (Engel et al., 2013; Schoffelen and Gross, 2009; Scholvinck et al., 2013). In particular, two distinct types of coupling have become prominent (Engel et al., 2013): The first arises from phase coupling between band-limited oscillatory signals, and the second is the result of synchronisation between the amplitude envelopes of band limited oscillations. Magnetoencephalography (MEG) has been used successfully to characterise these intrinsic mechanisms (Brookes et al., 2011a,b; Gow et al., 2008; Gross et al., 2001; Ioannides et al., 2000; Jerbi et al., 2007; Liu et al., 2010; Luckhoo et al., 2012; Marzetti et al., 2013; Nolte et al., 2004, 2008; Ramnani et al., 2004; Schlogl and Supp, 2006; Schoffelen and Gross, 2009; Tass et al., 1998; Wens et al., 2014), and much of the available evidence implies that envelope synchrony relates closely to the RSNs observed in fMRI. In fact, the spatial signatures of a number of fMRI based RSNs can be seen using MEG based envelope correlation metrics; this finding has now been observed in a number of studies (Brookes et al., 2011a,b; Hipp et al., 2012; Liu et al., 2010; Luckhoo et al., 2012; Wens et al., 2014). There is also emerging evidence showing that connectivity, as assessed by envelope synchronisation, is a dynamic process with significant non-stationarity observable in the resting state (Baker et al., 2012, 2014; Brookes et al., 2014; de Pasquale et al., 2010). Such MEG based measurements are not limited by the indirect nature of the haemodynamic response and therefore offer significant advantages in characterising transient connectivity. Overall, the apparent close relationship between neural oscillatory processes and RSNs, coupled with the promise of MEG based network measures to characterise dynamics, suggests that studies in this area offer an excellent opportunity to further our understanding of the dynamic connectome.

In this paper, we exploit the direct nature and good time resolution of MEG measured beta band neural oscillations to investigate transient functional connectivity in the sensorimotor network. We choose this network as it is one of the best characterised RSNs, whose morphology is open to direct interpretation. We hypothesise that the sensorimotor RSN described in the literature based on stationarity assumptions is, in fact, a temporally and spatially smoothed aggregate of multiple (more focal) transiently synchronising sub-networks (TSNs). To test this hypothesis, we combine a multivariate sliding window approach based upon canonical correlation analysis (CCA) (Barnes et al., 2011; Brookes et al., 2014; Hotelling, 1936; Soto et al., 2010) with vector quantisation (MacQueen, 1967) to generate a method to identify robustly occurring transient connectivity patterns. Applying this method to two separate datasets, we derive the spatial signatures of multiple TSNs occurring within the sensorimotor system. We show that these individual spatial signatures describe significantly more variance than any equivalent signature defined assuming stationarity. We go on to show that TSNs generalise across subjects and across independent experiments. Finally, we hypothesise that, on initiation of a motor task, efficient neural processing would favour recruitment of a specific set of sub-networks (that are also observable in resting data). We show that in spite of no significant change in overall connectivity between statically defined network nodes, specific sub-networks significantly increase their likelihood of occurrence during a task.

## Methods

### Data acquisition

Two separate MEG datasets were acquired. The first was designed as a ‘resting state’ recording with an intermittent self-paced motor response. The second comprised a cognitive task.

#### Dataset 1: self-paced motor

Ten volunteers (8 males, 2 females aged 25 ± 4 years (mean ± SD)) were asked to lie supine in the MEG system and execute a button press with the index finger of their non-dominant hand. Subjects were told that button presses should be repeated infrequently (approximately once every 30 s) for a total of 1200 s, and that they should not count in the period between presses. Ten right handed subjects were recruited. Button presses were recorded using a keypad.

#### Dataset 2: Sternberg working memory task

Eleven subjects (7 males, 4 females aged 31 ± 6 years (mean ± SD)) were recruited to this study. In the task, a single trial comprised presentation of two *example* visual stimuli (arbitrary black abstract shapes on a grey background, shown for 600 ms with 1 s between onsets); this was followed by a 6 s maintenance period and a third *probe* stimulus which was shown for a duration of 3 s. The subject was asked to respond, via right handed button press (index finger), if the *probe* stimulus matched either of the two *example* stimuli. A single block comprised three trials followed by a rest phase lasting 36 s; 15 blocks were presented to each subject. The probability of a target (i.e. that the probe matched one of the two example stimuli) was 0.5.

These two paradigms both contain a motor response (a button press). However, the difference between them allows contrast between simple motor action, infrequently performed during the resting state, and similar motor action set within a complex cognitive paradigm. It was reasoned that if TSN signatures were integral to sensorimotor processing, then equivalent TSNs should be observed for both tasks. In addition, the Sternberg task would allow investigation of TSN dynamics for fast and slow reaction times. Both experiments were approved by the University Of Nottingham School Of Medicine Ethical Committee.

All MEG data were collected using the synthetic third order gradiometer configuration of a 275-channel CTF MEG system (MISL, Coquitlam, Canada) at a sampling rate of either 1200 Hz (self-paced) or 600 Hz (Sternberg). Subjects were positioned supine. Prior to data acquisition, three head position indicator coils were placed on the head. These coils were energised periodically during data acquisition in order to localise the subject's head in the scanner. To facilitate co-registration of the MEG sensor geometry to brain anatomy, a 3D digitisation of the three fiducial points and the head surface was acquired using a Polhemus Isotrak digitiser system. Anatomical MR images were acquired using either a 3 T or 7 T Philips Acheiva MRI scanner at a voxel resolution of 1 mm^3^. Coregistration of MEG data to the anatomical MRI was completed by matching the digitised head surface (Polhemus) to the equivalent head surface extracted from the anatomical MRI.

### Data analysis

**Fig. 1:**
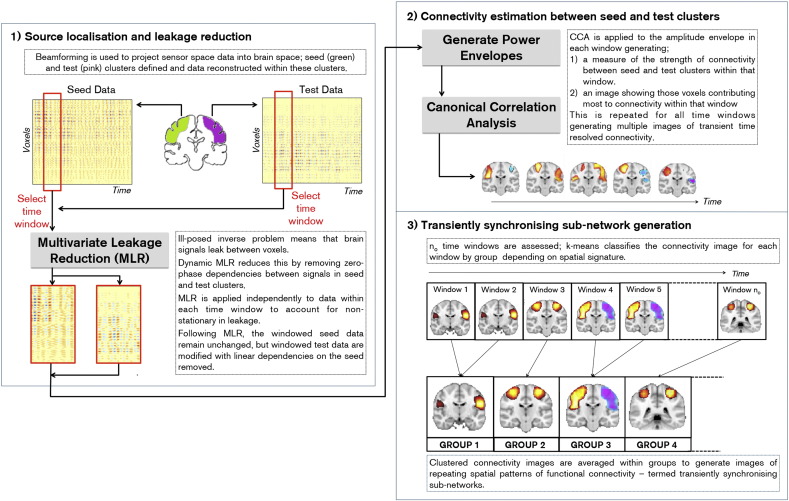
Schematic diagram showing the processing pipeline used to extract transiently synchronising networks.

A novel data processing pipeline was developed to image the hypothesised TSNs. This is shown schematically in Fig. 1. Functional connectivity was estimated as correlation between the amplitude envelopes of band limited neural oscillations in the left and right regions of the static sensorimotor network; this method was chosen over phase coherence due to its close relationship to RSN structure observed previously (Brookes et al., 2011b). Since previous studies show that sensorimotor network connectivity is strongest in the beta band (Brookes et al., 2011a, 2014; Hipp et al., 2012) analyses were limited to 13–30 Hz. Our technique used: 1) a spatial filter to project sensor space MEG data into brain space and dynamic multivariate leakage reduction to ameliorate the confounds of source space signal leakage (this is a critical step in order to prevent artefactual results — our technique for dealing with it is given in Supplementary material). 2) A sliding window canonical correlation analysis (CCA) to estimate the spatial signature of transient functional connectivity within each time window. 3) Vector quantisation (k-means clustering) to cluster connectivity images into repeating spatial patterns; it is these patterns which form our transiently synchronising sub-networks (TSNs). These steps are each described further below.

#### Source localisation and leakage correction

Source localisation was carried out using an adaptive beamformer (Robinson and Vrba, 1999; Van Veen et al., 1997). Covariance was computed in the beta band using a time window spanning the whole experiment (Brookes et al., 2008). Regularisation was applied to the data covariance matrix using the Tikhonov method, with a regularisation parameter set to ensure a condition number of 100. The forward model was based upon a dipole approximation (Sarvas, 1987) and a multiple local sphere head model (Huang et al., 1999). Dipole orientation was determined using a non-linear search for optimum signal to noise ratio (SNR). Source timecourses were computed at the vertices of a regular (8 mm) grid spanning the volume enclosed by the static sensorimotor network. The network mask was based upon an atlas derived using spatial independent component analysis applied to fMRI data (Filippini et al., 2009). The mask contains bilateral primary motor cortices as well as bilateral primary and secondary somatosensory cortices. Note that this spatial signature has featured in previously published studies (e.g. Brookes et al., 2011b; Filippini et al., 2009; Luckhoo et al., 2012) and represents a robust measure of the canonical static sensorimotor network. Beamformer estimated timecourses for all voxels within the mask were divided by hemisphere; a ‘seed’ cluster was defined, containing all voxels in the left hemisphere enclosed by the mask; likewise a ‘test’ cluster was defined containing all voxels in the right hemisphere enclosed by the mask (see Fig. 1).

Our subsequent analysis aims to measure inter-hemispheric connectivity between the seed and test clusters. The major confound of MEG connectivity measurements is signal leakage between source space timecourses (i.e. leakage between seed and test clusters) (Brookes et al., 2012, 2014; Hipp et al., 2012). This is a consequence of the ill-posed MEG inverse problem and typically results in artifactually inflated connectivity estimates. A simple method to reduce this leakage is based upon linear regression, which has been described in previous studies (Brookes et al., 2012; Hipp et al., 2012). However, here we note that our implicit assumptions of non-stationarity in functional connectivity bring with them implications for such standard methods to mitigate the effects of leakage. A difference between the two studies previously published is that Brookes et al. assumed stationarity, and performed a single leakage correction step for the whole dataset, whereas Hipp et al. proposed a dynamic approach correcting small time-windows individually. The advantage of the former is that the leakage correction will be more precise as it is based on more data. The advantage of the latter is that it will be robust for non-stationary data. In fact it can be shown (see Supplementary material) that when measuring functional connectivity across multiple time windows, if changes in variance in either a seed or test cluster timecourse are expected between windows, then dynamic leakage reduction is essential to ensure unbiased functional connectivity estimation. For this reason, in the present work, we used a dynamic multivariate regression approach to eliminate signal leakage between the seed and test clusters on a window by window basis.

#### Transient functional connectivity calculation via CCA

Following source localisation and leakage reduction, beamformer projected data for all voxels in the seed and test clusters were Hilbert transformed and their associated analytic signal computed. The absolute value of the analytic signal was then derived, generating timecourses of the envelope of beta oscillations for every voxel. These envelope timecourses were down-sampled temporally to 50 Hz to improve computational efficiency.

Canonical correlation analysis (CCA) (Hotelling, 1936) is a method to calculate statistical interdependencies between two multi-dimensional data matrices. CCA has been used extensively in previous MEG studies and complete descriptions can be found elsewhere (Barnes et al., 2011; Brookes et al., 2014; Soto et al., 2009, 2010). In the present context, CCA was applied across voxel timecourses to assess relationships between the beta envelopes in the seed and test clusters. A sliding window framework was used with canonical correlation measured independently within either 6 s windows (self-paced Study) or 3 s windows (Sternberg study). (The difference in window width across the two studies was to account for the shorter trial duration in the Sternberg task.) The sliding window allows a measure of temporal changes in correlation between data matrices. It is important to note that, since the columns of the seed and test data matrices comprise windowed beta amplitude envelopes from adjacent voxels, those columns are necessarily correlated due to the inherent smoothness of beamformer reconstruction. For this reason, prior to CCA, both matrices were decomposed using principal component analysis into four orthogonal features, thus allowing unambiguous assessment of the relationship between the seed and test clusters. A multivariate general linear model was then applied, describing temporal features in the test cluster as a linear mixture of features in the seed cluster. Appropriate analysis (see Supplementary material for details) then facilitates calculation of the optimal linear combination of features in seed and test clusters that maximise correlation. For any one time window, the canonical correlation coefficients estimate the strength of inter-hemispheric connectivity. More importantly, the canonical vectors give the optimal combination of features (hence voxels) that maximises connectivity. In this way, images can be generated showing which voxels contribute most to functional connectivity within any one time window. Sliding that window in time (using either 1 s steps (self-paced study) or 0.25 s steps (Sternberg study)) facilitates generation of many images, each showing the transient spatial signature of functional connectivity. These images were transformed spatially into MNI space using FLIRT in FSL (Jenkinson et al., 2012). Images were then concatenated across all 10 subjects for the self-paced study, and all 11 subjects for the Sternberg study.

In addition to the sliding window images, static images were also derived using the same CCA method, but with one single window spanning the entire duration of the experiment. These static images highlight voxels that contribute maximally to correlation between the seed and test clusters, over all time. They were transformed spatially into MNI space using FLIRT, averaged across subjects and used for direct comparison with the TSNs derived from the shorter sliding windows.

#### K-means clustering

Using the sliding window CCA approach, within a multi-subject dataset, several thousand images of connectivity are generated (specifically 11,940 and 25,272 for the self-paced and Sternberg studies respectively). This means that an automated process of grouping and classifying these images is desirable. K-means clustering (MacQueen, 1967) is a method of vector quantisation which has been used in recent fMRI experiments (Allen et al., 2014; Liu and Duyn, 2013) to detect repeating patterns of connectivity. If we assume a total of *n_o_* sliding windows across the experiment, then k-means partitions those *n_o_* connectivity images into *k* states. To do this, we first note that the images exist in an *f* dimensional space (where *f* represents the total number of voxels in the seed and test cluster combined). *k* points are then inserted into this space to form the centre of derived clusters and the k-means algorithm looks to minimise the within cluster sum of squares of Euclidian distance to the mean, over multiple iterations. Mathematically:

(1) $$\min_{S}\sum_{j=1}^{k}\sum_{I_{i}\in S_{j}}^{}{||I_{i}-\mu_{j}||}^{2}$$

where ***I***_i_ represents the *i*th connectivity image and ***μ**_j_* is the mean of the points in each projected group, ***S***_j_. Physically, these groupings represent images depicting similar functional connectivity patterns which consistently reoccur. We term these repeating patterns transiently synchronising sub-networks (TSNs). Note that in what follows we chose *k* = 8.

#### Testing TSN robustness

Our primary hypothesis is that the derived TSNs are spatially distinct (from each other and from the static network) and robust across subjects and datasets. The method outlined above offers a means to capture these spatial patterns. Statistical tests were then sought to validate their robustness. We devised three analyses:

#### Test 1 “Miss-a-TSN”

We first tested whether any of the 8 derived TSNs were redundant (i.e. not required to explain the data). To do this, a single CCA derived connectivity image was selected and its best fitting TSN selected. The percentage of variance in this image, explained by the best fitting (scaled) TSN, was then calculated. This process was repeated for all connectivity images within each subject, and the mean variance explained calculated. This analysis was repeated a further 8 times; on each iteration, a different TSN was removed from the basis set and replaced with the average network (generated as the mean across all connectivity images and subjects). We hypothesised that replacement of any one TSN with the average map would evoke a significant drop in variance explained. Significance was determined using a two-sided signed rank test of the null hypothesis that this difference originated from a distribution whose median is zero. The threshold for significance (p < 0.05) was Bonferroni corrected (to p_corrected_ < 0.0065) to account for multiple comparisons across the 8 TSNs. This test was carried out three times: On the self-paced dataset, on the Sternberg dataset, and finally on just the resting state phase of the self-paced dataset in order to determine whether any of the derived TSNs were only observable during the task.

#### Test 2 “Miss-a-subject”

We next assessed robustness across subjects by testing the hypothesis that TSN maps, derived via k-means, explained the data significantly better than the canonical (static) network map. For this purpose, we first selected a subject and removed their data from the full dataset; k-means was then run on the remaining (N − 1) subjects to derive a TSN basis set. A “sham” TSN basis set was also derived in which, rather than each connectivity image being assigned to a group via Eq. (1), it was assigned randomly. Note that these “sham” maps are computed without considering temporal structure in the measured connectivity (i.e. assuming stationarity), and for this reason we term them “static pseudo-networks”. This process generated two basis sets, both using N − 1 subjects. These two basis sets were then used to explain the variance in the remaining subject. We reasoned that if the TSN maps were robust across subjects then they would explain significantly more variance in the missing subjects' data than static pseudo-networks. This analysis was repeated for all subjects, generating a set of values of variance explained. We then tested whether TSN maps explained more variance than static pseudo-networks across N iterations of the missing subject.

#### Test 3 “Cross-dataset validation”

The above tests were run *within* datasets (i.e. either using Sternberg data only, or self-paced data only). However, if the TSNs derived using k-means are genuine transient networks that support sensorimotor function, then they should generalise to any task (or indeed the resting state). A cross-dataset validation was therefore performed in which we used the TSN basis set from the self-paced experiment to explain the Sternberg data, and vice versa. The TSN basis set from the self-paced data was taken along with an equivalent set of 8 static pseudo-networks. We reasoned that if the TSN maps were not robust, the TSN basis set from the self-paced study would explain no more variance in the Sternberg data than the static pseudo-networks. A null distribution was formed via generation of 2000 separate basis sets based upon different realisations of the static pseudo-networks, and we tested our hypothesis that the genuine TSN set (from the self-paced data) would explain significantly more variance in the Sternberg data than the sham basis-sets. This analysis was then reversed, and the Sternberg basis set used to explain the self-paced data, employing an identical methodology.

#### Task induced change in transiently synchronising sub-networks

Our secondary hypothesis was that, on task initiation, efficient neural processing would favour recruitment of a specific set of sub-networks. To measure how a task affected the likelihood of occurrence of a network, for each TSN we first constructed a binary timecourse. This was computed across all task trials and subjects and was based on k-means grouping; it contained a 1 if the current window belonged to the TSN group of interest, or a 0 otherwise. This vector was summed across task trials (over all subjects) and divided by the total number of trials; the result is a timecourse showing the probability of a specific TSN being selected for any time window within a trial (see Supplementary Fig. S1). Dividing these timecourses by the overall fraction of windows classified in the group enabled measurement of the fractional change in probability of observing any one network, at any time point within a trial. A deflection in these timecourses would highlight that the TSN in question was more, or less likely to be observed within that time window.

Finally, a method was devised to confirm that any observed deflection in the probability timecourses was due to localised changes in functional connectivity within the TSN in question. This was achieved via a ‘point-to-point’ transient connectivity analysis. To compute point-to-point connectivity, firstly, two points (a seed and test) were selected based upon the peaks in a TSN map; source timecourses were then estimated using the beamformer as described above. A sliding window was allowed to shift across the timecourses and a dynamic (univariate) leakage reduction applied within each window. Following leakage reduction, the amplitude envelope of both the seed and test timecourses (within each window) was computed via Hilbert transformation and connectivity estimated, via (univariate) correlation, within each window. These connectivity timecourses were averaged across task trials within each individual subject. To allow for changes in the temporal scale of functional connectivity, this process was repeated for window widths ranging from 2 s to 48 s, in the case of the self-paced motor study, and 2 s to 10 s in the case of the Sternberg study. (Note such variation in window widths is impractical for CCA due to computational load.) To determine the statistical significance of task-induced changes in connectivity, the mean variances explained in windows encapsulating the event of interest (the button press) and for windows only capturing rest, were computed and the difference calculated. This was repeated for each subject individually and statistical significance of the difference in measured connectivity between task and non-task windows was computed.

## Results

### Transiently synchronous sub-network generation and evaluation

**Fig. 2:**
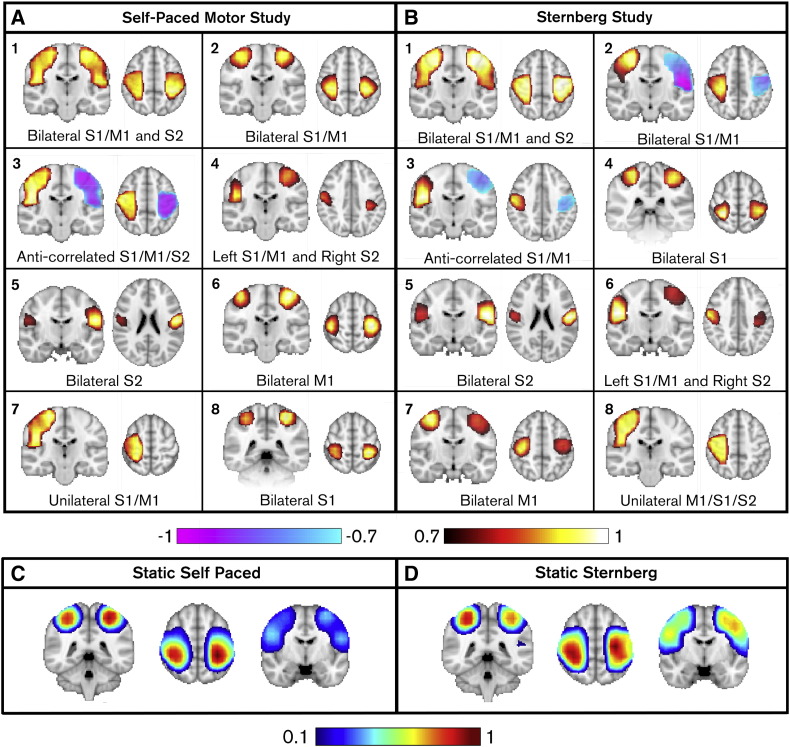
Transiently synchronous sensorimotor sub-networks generated using two independent datasets. The left hand side (A) shows a 10-subject dataset in which participants executed an infrequent self-paced button press. The right hand side (B) shows an 11-subject dataset in which participants were involved in a Sternberg working memory task. Note the equivalence of the observed transient connectivity images. Note also the highly focal nature of the spatial topographies. (C–D) Static connectivity images generated using a window spanning the entire experiment.

Fig. 2 shows the TSN maps for the self-paced (A) and Sternberg (B) tasks. Our hypothesis that multiple, spatially distinct and focal TSNs would be observed is supported by Fig. 2, which shows that spatial patterns representing transient functional connectivity differ in time. In the self-paced dataset (Fig. 2A), *TSN1* covers bilateral primary motor and sensory cortex and extends inferior to S2. *TSN2* only covers primary M1 and S1 regions whilst *TSN5* captures only bilateral S2. *TSN6* and *TSN8* separate anterior and posterior sensorimotor regions: assessment of the peak locations reveals MNI coordinates of (− 36,24,60) mm and (40,− 22,60) mm for *TSN6* which equate to the left and right precentral gyri (Brodmann area 4). MNI coordinates for *TSN8* were (30,− 38,58) mm and (34,− 30,60) mm; the peak in right hemisphere is centred on postcentral gyrus (Brodmann area 3) and the peak in left hemisphere is less than 1 voxel from the postcentral gyrus (Brodmann area 3). This evidence shows that bilateral sensory and motor cortices form independent transient networks and our method facilitates their separation. In addition to positive correlations, negative correlations are also observed in *TSN3*, showing that the method captures windows in which the beta envelopes in the left and right sensorimotor strips are anti-correlated. Finally, *TSN4* highlights a spatially asymmetric TSN (left M1/S1 and right S2) and *TSN7* depicts a unilateral response. Results for the Sternberg (Fig. 2B) task are similar (Fig. 2A) and again include anti-correlated networks (*TSN2* and *TSN3*), bilateral S2 (*TSN5*) and a spatially asymmetric network (*TSN6*) covering left M1/S1 and right S2. Motor and sensory cortices (*TSN7* and *TSN4*) are again separated. In addition to the clear similarity across these two completely independent experiments, note also the highly focal nature of the TSN maps.

For comparison, Figs. 2C and D show the static connectivity images generated using the self-paced and Sternberg datasets respectively. These images were generated using the same CCA approach, but with a single time window capturing the entire experiment. In contrast to the TSN maps, the static map is less spatially specific. Whilst clear foci are observed, they appear to spread across primary sensory and motor regions, and the map extends down to S2 (albeit at a lower threshold). Most importantly, the subtle spatial dynamics observed in the TSN measurements are missed by the static approach.

The robustness of each individual TSN was tested using a “miss-a-TSN” analysis. We tested how much variance in the *n_o_* connectivity images could be explained by our TSN maps, and whether replacing a single TSN with a static network caused a significant drop in the variance explained. The 8 TSNs in Fig. 2A explained 71 ± 3% of variance in the self-paced connectivity images. Replacing a single TSN with the static network gave rise to a significant (p_corrected_ < 0.05) drop in explained variance for 6 of the 8 TSNs. The exceptions were TSN1 (p_corrected_ = 0.08) and TSN7 (no trend). In the case of TSN1, the spatial signature is similar to the canonical network and it is unsurprising that replacement evokes no significant drop in variance explained. TSN7 is unilateral and reflects close to zero connectivity, meaning that the canonical correlation between cortices when this mode was detected was 0.06 ± 0.05 (considerably lower than all other modes which average > 0.2). Equivalent analysis was applied to the resting state phase of the self-paced data; i.e. within data windows not capturing the infrequent motor task. Results were identical, showing that the TSNs are also a feature of resting state data. Likewise, the 8 maps in Fig. 2B explained 73 ± 1% of variance in the Sternberg images and again, replacing a TSN with the static network gave rise to a significant (p_corrected_ < 0.05) drop in explained variance for 6 of the 8 TSNs. Once again exceptions were TSN1 (which resembles the static map) and the unilateral network (TSN8).

Robustness of TSNs over subjects was tested by a “miss-a-subject” analysis. Here, vector quantisation was applied to the connectivity images as before, but with a single subject missing. The resulting TSN maps were then used to explain variance in that missing subject. Running vector quantisation with a subject missing made little difference to the TSN morphology. In the self-paced data, TSN maps on 9 subjects were 99.6 ± 0.4% correlated with the maps in Fig. 2A (10 subjects). For the Sternberg data, TSN maps made using 10 subjects were 99.8 ± 0.2% correlated with those in Fig. 2B (11 subjects). The TSN maps generated with a missing subject explained 69 ± 3% of variance in the omitted subjects' data in the self-paced experiment, and 72 ± 2% in the Sternberg experiment. Replacement of the TSNs with an equivalent number of static pseudo-networks gave rise to a significant drop in variance explained from 69 ± 3% to 47 ± 7% for the self-paced data (p = 0.002) and from 72 ± 2% to 39 ± 2% for the Sternberg data (p = 0.001). This confirmed not only robustness over subjects, but also that the TSNs were a significantly better representation of transient connectivity than canonical static networks.

As a final test, we reasoned that if TSN maps represent transient networks that are a fundamental component of sensorimotor processing, then they should generalise to any task. Specifically a TSN basis set from task A should better explain the connectivity in task B than any static network. We therefore employed our cross dataset validation, using the self-paced TSNs (Fig. 2A) as training data to predict the Sternberg connectivity images, and the Sternberg TSNs (Fig. 2B) as training data to predict the self-paced connectivity images. These results were compared to equivalent within dataset measurements. 73 ± 1% of variance in the Sternberg data was predicted by the Sternberg derived TSNs, and this was reduced marginally to 71 ± 2% when using the self-paced TSNs as training data. Likewise, 71 ± 3% of variance in the self-paced data was explained by the self-paced TSN maps, which was reduced to 69 ± 2% when using the Sternberg TSN maps as training data. The maximum variance explained in the Sternberg data across 2000 iterations of static pseudo-networks was 40.8%. Similarly, the maximum variance explained in the self-paced data across 2000 iterations of static pseudo-networks was 41.7%. This shows clearly that TSNs, even from a completely independent dataset, represent a better model of transient connectivity than the canonical network.

**Fig. 3:**
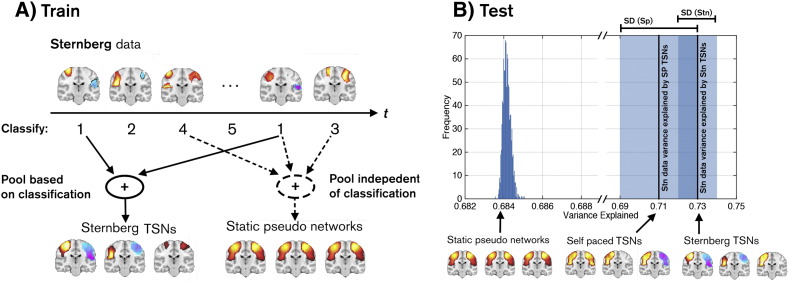
(A) Schematic representation of the process to generate both the real TSNs, and a series of static pseudo-networks to test the null hypothesis. Real TSNs are generated based on the state allocation of individual connectivity images from the k-means clustering process, whilst for the pseudo-static networks, states are assigned assuming stationarity. (B) The resulting variance explained in the Sternberg connectivity data by 2000 permutations of the static pseudo-networks (histogram) and the TSNs from both the self-paced and Sternberg datasets. Note that using self-paced rather than Sternberg TSNs to explain the Sternberg data does not result in a significant drop in variance explained, thus highlighting robustness of the TSN maps over the experiments. Note also that the null hypothesis is rejected.

A post-hoc concern was that the significant differences in variance explained between TSNs and static pseudo-networks may be driven entirely by the transient anti-correlated networks, or by those networks deemed unimportant by our ‘miss-a-TSN’ analysis (e.g. TSN1 and TSN7 in Fig. 2A; see above). For this reason a new set of static pseudo-networks were generated: this new training set contained a mix of the TSN maps from the real basis set, and pseudo-networks (again generated via random assignment of group number to the remaining training data). We found that TSNs 2, 4, 5, 6 and 8 in Fig. 2A explained significantly more variance in the Sternberg data than equivalent pseudo-networks, and likewise TSNs 4, 5, 6 and 7 in Fig. 2B explained significantly more variance in the self-paced data than equivalent static pseudo-networks (see Fig. 3).

The above analyses show that the canonical sensorimotor network, far from being a single entity, is composed of multiple transiently synchronous (and spatially focussed) patterns of functional connectivity where the involved nodes rapidly change their connectivity — from being positively correlated, uncorrelated to strongly anti-correlated. These patterns explain MEG connectivity data significantly better than static networks and are not only robust across subjects, but are also reproducible in two independent experiments.

### Task induced change in functional connectivity

**Fig. 4:**
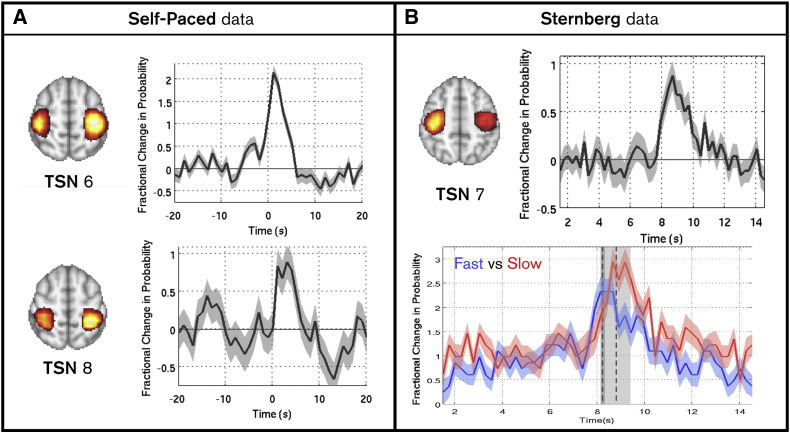
Task induced fractional change in TSN probability. Panel (A) shows the self-paced data. Note that only the two networks that exhibit a significant task induced change are shown. TSN6 covers the bilateral motor cortex and TSN8 captures the bilateral sensory cortex. Panel (B) shows the Sternberg data. The upper panel shows the trial average occupancy change for TSN7. The lower panel contrasts trials with a fast reaction time (8.21 ± 0.09 s, blue trace) with trials with a slow reaction time (8.78 ± 0.59 s, red trace) (see Supplementary Figs. S2 and S3 for further results).

Timecourses were generated to measure task induced changes in the probability of observing a specific TSN. An increase in these timecourses means that a TSN is more likely to be observed at a specific time point; a decrease means the TSN is less likely to be observed. Fig. 4A shows the examples for self-paced data: timecourses represent the fractional change in probability for two selected TSNs. TSN6, which covers bilateral M1, exhibits a significant (p < 0.05) change around the time of the button press showing that we are ~ 200% more likely to observe this TSN during a single finger movement (with one hand), compared to rest. Likewise TSN8, which covers bilateral sensory cortex also exhibits a significant (p < 0.05) task induced response. Similar results were observed for the Sternberg data and are shown in Fig. 4B. Here TSN7 (again bilateral M1) exhibits a significant (p < 0.05) change in occupancy around the time of the button press ($\bar{t}$ = 8.41 s). The lower panel also shows probability timecourses, but contrasts trials with a fast reaction time (8.21 ± 0.09 s), against trials with a slow reaction time (8.78 ± 0.59 s). Note the difference in time to peak and longevity of response. These results support the hypothesis that on task initiation the relative occupancy of TSN states is altered.

**Fig. 5:**
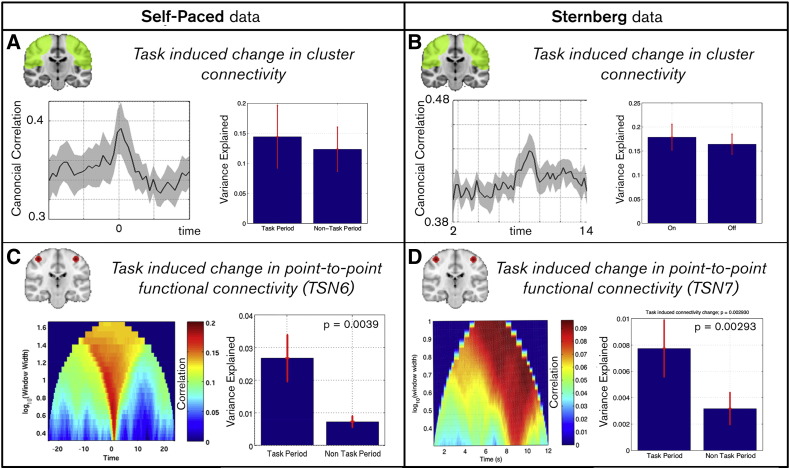
Task induced change in functional connectivity at differing spatial and temporal scales. (A/B) Connectivity between clusters. Timecourses show the trial averaged response whereas bar charts show the mean variance explained in the test cluster by the seed cluster, in windows capturing the button press compared to those not capturing the button press. (C/D) Univariate connectivity between point locations. Pairs of voxels were selected based upon TSN6 (self-paced) and TSN7 (Sternberg). In the left hand plot the x-axis shows the time relative to the button press, the y axis shows the log_10_(window width) and the colour shows the strength of connectivity (correlation between the Hilbert envelopes of beta oscillations, within the window). The bar graphs show the variance explained by the seed location at the test location in windows encapsulating or not encapsulating the button press.

Finally, Fig. 5 probes the spatial and temporal scales of task induced change in functional connectivity. Figs. 5A and B show the trial averaged canonical correlation between clusters covering the sensorimotor network. The timecourses shown represent change in total inter-hemispheric functional connectivity within the sensorimotor system. Note that in both the self-paced and Sternberg experiments, a transient increase in connectivity between clusters is observable around the time of the button press. However, this increase is modest, as evidenced by the bar charts which show mean connectivity between clusters in windows capturing the button press compared to those capturing resting state. In the self-paced data, the variance explained in the test cluster by the seed was greater by 11 ± 9% in the windows containing the button press, whilst in the Sternberg data the same measure increased by 9 ± 3%; in both cases the change failed to reach statistical significance across subjects. Figs. 5C and D show measured task induced change in functional connectivity between point locations selected on the basis of the TSN maps. Specifically, results show functional connectivity between primary motor areas (TSN6 for self-paced data and TSN7 in Sternberg data). Point-to-point connectivity is assessed using a univariate sliding window approach. Multiple window widths are shown collectively in the figure. Connectivity is averaged over task trials; the x-axis shows time relative to the button press, the y-axis shows log_10_(window width) and the colour shows connectivity strength (windowed correlation between beta envelope timecourses). The bar graphs show variance explained by the seed location at the test location. Windows encapsulating the button press are contrasted with those not encapsulating the button press. Figs. 4 and 5 are complementary. The increase in occupancy of specific TSNs during motor behaviour (Fig. 4) shows that efficient neural processing requires dominance of a specific sub-network to support movement. During movement, sensorimotor network functional connectivity is thus dominated by a small number of highly focal networks. This is evidenced by the increased functional connectivity between bilateral M1 in Figs. 5C and D. However, this focal increase has relatively little effect on inter-hemispheric connectivity within the wider network (Figs. 5A and B).

## Discussion

Using a new method for imaging transient patterns of functional connectivity, we have shown that the static metrics most often used to characterise coupling between network nodes fail to provide a complete picture of the complex spatio-temporal dynamics within the network they are attempting to describe. By exploiting the excellent time resolution of MEG, with advanced leakage reduction and multivariate connectivity modelling, we were able to show that the static sensorimotor network can be decomposed into multiple dynamically changing sub-networks. These sub-networks have been observed without the use of statistical priors and with unsurpassed spatiotemporal accuracy. We have shown that these TSNs are not only a common feature across subjects, but are also a common feature across completely independent multi-subject experiments. Indeed the evidence is that the commonly observed static network oversimplifies the ground truth: our data show clearly that individual areas of the larger network progress through stages of highly correlated, uncorrelated and even strongly anti-correlated activity. In addition we have shown that TSNs are a consistent feature of the resting state, and that task initiation serves to bias the likelihood of a particular TSN being recruited.

The observed spatial patterns represent physiologically interpretable networks of connectivity. Most noteworthy, our results show that, even outside a task, functionally specific and spatially focal brain areas can be extracted blindly. In some cases broad complexes of bilateral homologous regions were identified: for example in both studies the most commonly occurring TSN comprised bilateral M1 and S1, extending down to bilateral S2. Other networks revealed highly focal complexes, including bilateral primary motor area (M1), bilateral primary somatosensory area (S1) and bilateral secondary somatosensory area (S2). In particular, the clear separation of motor (M1) and somatosensory (S1) cortices into two separate networks, despite these regions being separated by only a few millimetres, shows the spatial accuracy of the technique. The extraction of such neuroanatomical detail from MEG data is rare, particularly in the resting state. The existence of anti-correlated networks in both tasks suggests a transiently occurring antagonistic relationship between beta envelopes within some time windows. Such anti-correlation may result from random mind-wandering; for instance it is known that attending to a particular location in the body causes anti-correlated shifts in the amplitude of somatosensory beta band oscillations within the two hemispheres (Bauer et al., 2012; van Ede et al., 2014). Likewise imagining movement, or even specific body parts can cause similar effects (Brinkman et al., 2014; de Lange et al., 2008). The existence of an asymmetric network (covering right S2 and left S1/M1) is also interesting. It is known that transient connections between left M1/S1 and right S2 occur during tactile stimulus processing (Simoes et al., 2003) and that connectivity between S1 and S2 has been associated with subjective perception (Ploner et al., 2009). This observation is therefore physiologically interpretable.

An important point is that, although the results presented were obtained in the context of two disparate paradigms, neither were “pure resting state”. In our self-paced task, participants were pressing a button every 30 s but for the remainder of the period participants remained at rest. This allowed for confirmation of the existence of TSNs with the brain (apparently) at rest, and simultaneously enabled validation of our methodology for robustly uncovering task induced temporal fluctuations of sensorimotor sub-networks. This said, it is conceivable that differences may result between the resting phase of our self-paced task, and ‘pure’ resting state data (in which subjects lie in a scanner and “think of nothing”). To account for this limitation, our methodology was also applied to 10 minute “pure rest” recordings in 10 subjects (for results see Supplementary Fig. S4). Once again TSNs were largely similar with our methodology separating M1, S1 and S2 as well as identifying anti-correlated and as asymmetric networks. This, coupled with our statistical (“miss-a-TSN”) analyses shows convincingly that the TSNs presented are a consistent feature of the resting state sensorimotor system.

Our secondary hypothesis was that, on initiation of a motor task, efficient neural processing would favour recruitment of a specific set of transiently synchronising sub-networks. We have shown that functional connectivity between sub-network nodes in bilateral M1 consistently and transiently changes around the time of overt motor behaviour. This is evidenced by i) an increase in occupancy of the M1 TSN (Fig. 4) and ii) an increase in transient univariate connectivity measured between bilateral M1 (Figs. 5C and D). Interestingly, these highly focal changes do not result in a drastic overall change in inter-hemispheric functional connectivity within the sensorimotor network (Figs. 5A and B). At a practical level this is important: if region to region connectivity is measured the overall effect of a task may be ‘washed out’ across voxels. However, if point-to-point connectivity is assessed, this will likely result in significant task induced change. However, the latter necessarily relies on a-priori selection of the precise points to be considered; our TSN analysis, for the first time, offers a principled means to assess task induced changes in network connectivity without such confounds. At a more theoretical level this finding offers an interpretation of task induced connectivity. Fig. 2 shows that sensorimotor network connectivity is maintained via several TSNs and, at rest, all of these spatial signatures, including those identified as relating to movement contribute to the high level of functional connectivity between the left and right sensorimotor strip. We speculate that active processing of a motor response simply involves the transient reorganisation of the resting state TSNs. This implies that active processing is not an additive process, but rests on simple spatial reorganisation of the wider sensorimotor network. Such a model explains the differences in connectivity across spatial scales shown in Fig. 5 and should be further tested in future studies of task induced functional connectivity change using the same methodology.

### Technical considerations

The methodology that we introduce is critically dependent on multiple factors, including selection of the underlying source localisation algorithm, and selection of a parameter set for the CCA and k-means algorithms. These important factors warrant further discussion.

At the core of the method is the beamformer spatial filter, however it is important to understand that any source localisation technique (e.g. Minimum Norm, dSPM) could be used. It is well known that beamforming suppresses spatially separate but temporally correlated sources (Brookes et al., 2007) and superficially this may appear as a confound for connectivity metrics which actively seek temporal correlation between sources. However, the beamformer has been used successfully in multiple studies of functional connectivity (Brookes et al., 2011a,b; Hipp et al., 2012), and could be argued to be the source localisation method of choice for such measurements. To understand this, first note that for beamformer suppression to take place, zero time lagged correlation must exist between source signals, whereas our metrics of connectivity measure temporal correlation between oscillatory envelopes. Importantly, two envelopes can be perfectly correlated whilst the underlying signals remain orthogonal. In fact, zero-time-lag correlated signals potentially reflect source leakage; indeed our leakage reduction algorithm actively aims to remove such effects. It therefore follows that, rather than the beamformer suppression of correlated sources acting as a confound, it actually helps to suppress leakage. Beamforming also offers excellent interference rejection properties and good spatial resolution, both of which are attractive when measuring functional connectivity. These important points should be noted when choosing underlying source reconstruction methodology.

As with all neuroimaging methods, our technique requires selection of a parameter set, with parameters including the number of eigenmodes *d*, the time frequency window size, the cluster location/extent and the number of spatial modes, *k*. There is no hard rule for selection of these parameters, and they will ultimately depend on the scientific question to be addressed. However, technical limitations also underlie parameter selection and this deserves discussion. In the present work we aimed to identify multiple TSNs in the sensorimotor system, with regions of interest covering bilateral sensory cortex and motor cortices. The previously published work in this area allowed for narrowing of the frequency range of interest to the beta band. This meant that both cluster size/location and frequency range were set directly by the neurophysiological question of interest. Selection of the number of eigenmodes, *d*, involves a direct trade-off between the cluster size and the time frequency window size used in the sliding window analysis. *d* must be sufficiently high to allow for expression of all of the signal features observable within a cluster (ideally *d* would be equal to the number of resolution elements within the cluster). Practically this can be quantified by calculating the variance in the original data explained by features retained; here selecting *d* = 4 explained 77 ± 3% of data (average across all subjects and clusters). Selection of d also impacts on the time window selection. As a rule of thumb, one requires more than 4*d* independent temporal observations within the sliding time window for the multivariate test to be reliable (i.e. if *d* becomes large then the number of time points in the window must also be large). Here we chose *d* = 4 and we employed a minimum window width of 3 s: The number of independent time samples in an envelope signal can be approximated as *B*_*W*_∆*t* where *B*_*W*_ represents the signal bandwidth (17 Hz for the 13–30 Hz beta band) and ∆*t* is the window width. This means that within any one 3 s time window we have ~ 51 independent time points. For the present paper this is well above the lower limit of 4*d* = 16, in order to ensure reliability of the test. In principle, using the data presented here, a 1 s time window should be possible. However, if the frequency band was reduced (e.g. if we looked at the alpha band where the bandwidth is ~ 5 Hz) then either d must be reduced or the time window increased. Finally, the number of states to extract via k-means (*k*) must also be selected. Here we chose *k* = 8, which was set empirically. Whilst this potentially reflects a limitation, such empirical selection not uncommon and is analogous to methods employing ICA, in which number of components is often set by visual inspection of the output. Most importantly, using our ‘miss-a-TSN’ analysis, the contribution of each TSN to the overall explanation of variance in the connectivity images was assessed quantitatively. In this way, we were able to show whether removal of specific TSNs impacted significantly the variance explained in connectivity images. This analysis is key to avoid over fitting and should be undertaken by researchers using this technique.

As a final note, we should mention that in this paper, following CCA we extract only the first of d eigenmodes of connectivity to take forward to the subsequent k-means analysis. However, this reflects a potential limitation. For any single window there are *d* − 1 further modes available that are (currently) ignored. These extra eigenmodes correspond to extra orthogonal mixtures of the features in the seed and test clusters that may also describe transient networks. It is possible (even likely) that the TSN maps shown in Fig. 2 might also be represented in these higher order eigenmodes. For example, if a bilateral S2 network in window 1 becomes dominated by a bilateral S1 network in window 2, it is likely that the S2 network has not ‘disappeared’, but rather persists at a lower level of functional connectivity and may well be represented by the extra eigenmodes. Harnessing these modes, and incorporating them into k-means clustering, would not only generate further insights and possibly allow tracking of individual transiently synchronising networks in time, but may also increase the effective number of averages contributing to the TSN maps, hence improve signal to noise. Future studies may wish to account for this.

## Conclusion

Resting state networks are of fundamental importance to neuroscience with evidence suggesting that they are integral to brain function and perturbed in pathology. However, the temporal dynamics of the functional connectivities underlying RSN structure are poorly understood. We have presented a framework to further our understanding of RSN dynamics. Using MEG, we have shown that the canonical sensorimotor network can be decomposed into transiently synchronising sub-networks, recruitment of which depends on current mental state. These sub-networks are highly focal, show rich temporal dynamics, and the interpretation is that the larger canonical network reflects only a temporal aggregate of transient functional sub-networks. The methodology developed opens new frontiers to study RSN dynamics; for example our technique could be applied to study other RSNs (e.g. DMN), between network connectivity, other frequency bands, different tasks, and patient populations. In this way, we have provided a new dimension in which to reveal the spatial, temporal and spectral signature of the human connectome in health and disease.
