# Supplementary material for: Dynamic recruitment of resting state sub-networks
Source: Neuroimage. 2015 Jul 15;115:85–95. doi: 10.1016/j.neuroimage.2015.04.030 (PMC4573462; doi:10.1016/j.neuroimage.2015.04.030)
Supplement: Supplementary file 1 — Supplementary material. [file mmc1.docx]

Dynamic recruitment of resting state sub-networks:

SUPPLEMENTARY MATERIAL

George C. O’Neill, Markus Bauer, Mark W. Woolrich, Peter G. Morris, Gareth R. Barnes and Matthew J. Brookes

1. **SUPPLEMENTARY METHODOLOGY:**

In this section, we provide a more detailed methodological description of Canonical Correlation Analysis (CCA) used in the main manuscript, and a graphical description of the TSN timecourse generation.

***Imaging transient functional connectivity using CCA***

Consider 2 matrices: $\boldsymbol{X}$ represents the oscillatory envelopes from the seed cluster within a single window (dimensions $m \times\delta$) and $\boldsymbol{Y}$ represents the oscillatory envelopes from the test cluster from the same window (dimensions $n\boldsymbol{\times}\delta$). Here, $m$ and $n$ are the number of voxels in the seed and test clusters respectively and $\delta$ is the number of time samples within a single window. We first note that the columns of both **X** and **Y** both represent envelope timecourses from neighbouring voxels, and as such are necessarily correlated due to the inherent smoothness of beamformer reconstruction. For this reason, those matrices are projected onto a reduced-dimension eigenspace to facilitate orthogonalisation, which is a requirement if CCA is to be applied unambiguously. Orthogalisation is accomplished by eigenvalue decomposition of the covariance of $\boldsymbol{X}$ and $\boldsymbol{Y}$; specifically we let $\boldsymbol{X}^{T}\boldsymbol{X =}\boldsymbol{U}_{\boldsymbol{X}}\boldsymbol{S}_{\boldsymbol{X}}\boldsymbol{U}_{\boldsymbol{X}}^{T}$ and $\boldsymbol{Y}^{T}\boldsymbol{Y =}\boldsymbol{U}_{\boldsymbol{Y}}\boldsymbol{S}_{\boldsymbol{Y}}\boldsymbol{U}_{\boldsymbol{Y}}^{T}$ **.** The columns of ***U_X_*** (or ***U_Y_***) contain the eigenvectors of the covariance $\boldsymbol{X}^{T}\boldsymbol{X}$ (or $\boldsymbol{Y}^{T}\boldsymbol{Y}$) and ***S_X_*** and ***S_Y_*** are diagonal matrices containing the associated eigenvalues. **U_X_** and **U_Y_** are truncated such that only the first $d$ columns are retained (which relate to the highest eigenvalues; in this study *d = 4*). Orthogonalised matrices, $\boldsymbol{X}_{o}$ and $\boldsymbol{Y}_{o}$, both with dimension $d \times\delta$ and whose columns are linearly independent, are generated as $\boldsymbol{X}_{o}\boldsymbol{=X}\boldsymbol{U}_{\boldsymbol{X}trunc}$ and $\boldsymbol{Y}_{o}\boldsymbol{=Y}\boldsymbol{U}_{\boldsymbol{Y}trunc}$. This removes any temporal correlation across features and facilitates unambiguous assessment of the relationship between the seed and test clusters. Using a multivariate general linear model:

$\boldsymbol{Y}_{o}=\boldsymbol{X}_{o}\boldsymbol{\beta}+\boldsymbol{\epsilon}$ [1]

Here, $\boldsymbol{\beta}$ is estimated as $\hat{\boldsymbol{\beta}}=\boldsymbol{X}_{\boldsymbol{o}}^{+}\boldsymbol{Y}_{\boldsymbol{o}}$ (where a superscript + indicates a Moore-Penrose pseudoinverse) and represents the matrix of regression coefficients best predicting ***Y_o_*** from ***X_o_***. The error term, $\boldsymbol{\epsilon}$, represents the unexplained signal in ***Y_o_*** and would be large if no relationship (connectivity) existed between the seed and test clusters. The ratio of explained to unexplained covariance is given by:

$\boldsymbol{D=}\frac{\left( \boldsymbol{X}_{o}\boldsymbol{\beta} \right)^{T}(\boldsymbol{X}_{o}\boldsymbol{\beta})}{\epsilon^{T}\epsilon}$ [2]

And eigenvalue decomposition of $\boldsymbol{D}$ generates:

$\boldsymbol{D=A}\boldsymbol{S}_{\boldsymbol{D}}\boldsymbol{A}^{T}$ [3]

Here, the columns of $\boldsymbol{A}$ are the canonical vectors, which give the best combination of features in $\boldsymbol{Y}_{o}$ that explain the features of $\boldsymbol{X}_{o}$. Similarly, the canonical vectors for $\boldsymbol{X}_{o}$ can be calculated as

$\boldsymbol{B}=\boldsymbol{\beta A}$. [4]

The canonical vectors **A** and **B** can be used calculate the canonical variates; these comprise the composite timecourses; that is to say the weighted sum of the columns of $\boldsymbol{X}_{o}$ and $\boldsymbol{Y}_{o}$ that maximise correlation in the window of interest. The canonical variates in $\boldsymbol{X}_{o}$ are given by and the canonical variates in are given by . It then becomes possible to compute the canonical correlation coefficients as:

[5]

The matrix has dimension and the elements represent correlation between the various eigenmodes of correlation. The eigenmodes are orthogonal and so all off-diagonal elements of are zero. The diagonal elements comprise a set of canonical correlation coefficients, one for each of the $d$ eigenmodes. A set of *d* canonical correlation coefficients exists for each time window in the sliding window analysis (which we denote by subscript *i*). The magnitude of correlation represents the strength of connectivity in that window. The canonical vectors, **A** and **B**, can also be used to assess which features (and therefore which voxels) contribute to connectivity. The canonical vectors (in feature space) are projected into voxel space thus:

$\boldsymbol{I}_{\boldsymbol{X}}\boldsymbol{=}\boldsymbol{U}_{\boldsymbol{X}}^{T}\boldsymbol{B}$ **;** $\boldsymbol{I}_{\boldsymbol{Y}}\boldsymbol{=}\boldsymbol{U}_{\boldsymbol{Y}}^{T}\boldsymbol{A}$ [6]

$\boldsymbol{I}_{\boldsymbol{X}}$ and $\boldsymbol{I}_{\boldsymbol{Y}}$ are the resulting images (similar to factor loadings in principle component analysis), representing the weighted sum of voxels in the seed and test regions that maximise correlation between the two clusters. $\boldsymbol{I}_{\boldsymbol{X}}$ and $\boldsymbol{I}_{\boldsymbol{Y}}$ are combined to generate:

$\boldsymbol{I}_{\boldsymbol{i}}\boldsymbol{=}\left[ \boldsymbol{I}_{\boldsymbol{x}}\boldsymbol{,}\boldsymbol{I}_{\boldsymbol{y}} \right]$ [7]

which gives a single image of connectivity. One of these images is generated per window (again indexed *i*). The strength of the connectivity in that window is given by the associated canonical correlation coefficient. Note that in principle a single image and correlation coefficient can be computed for each of the $d$ eigenmodes. However in this paper we consider only the first eigenmode, meaning that for any one window, is a scalar and $\boldsymbol{I}_{\boldsymbol{i}}$ a vector.

***TSN timecourse construction***

Figure S1 shows, schematically, how the probability timecourses for any on one transiently synchronising sub-network, are constructed. This is the bases of the timecourses in figure 4.

**
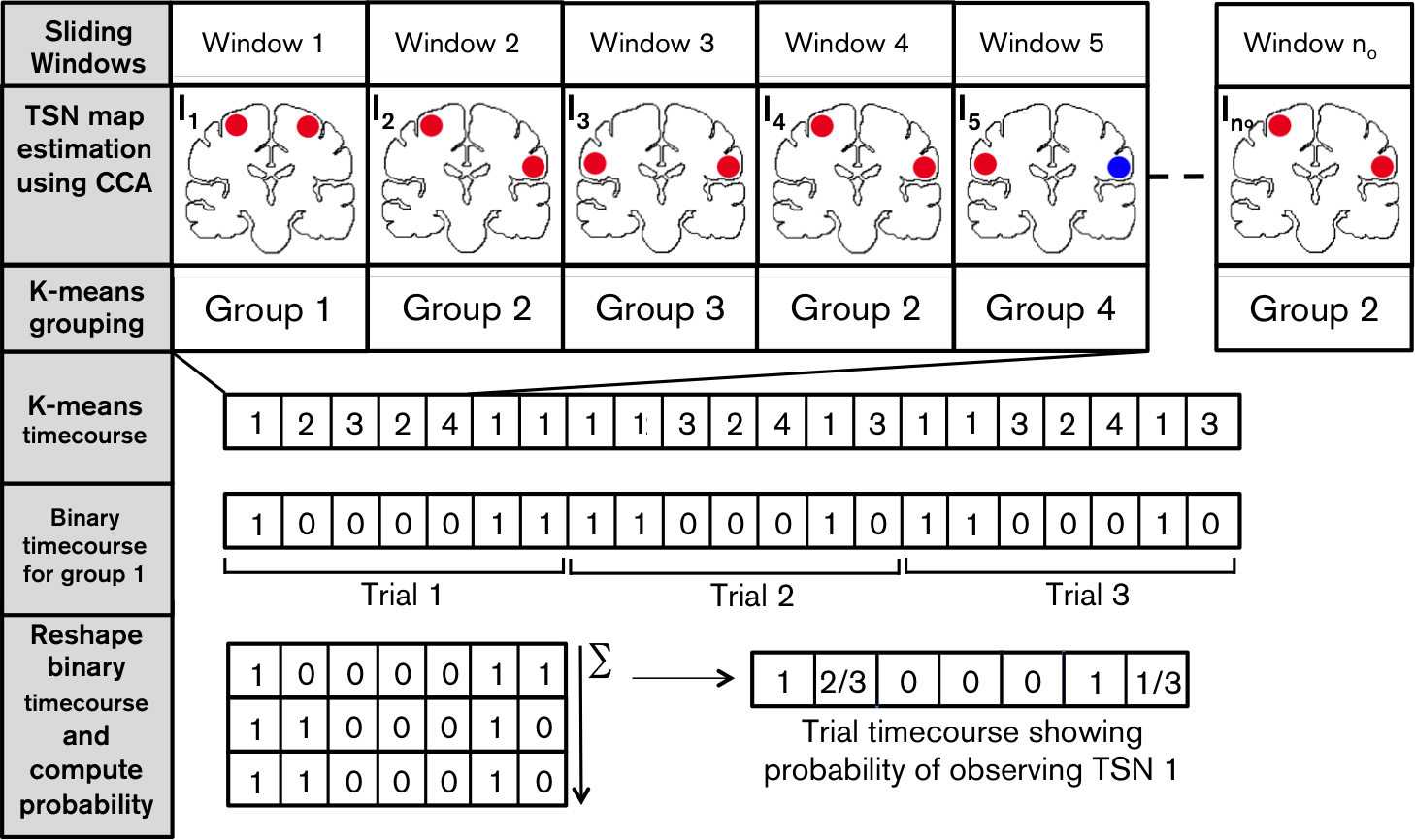
**

**Figure S1:** Schematic showing construction of TSN probability timecourses.

1. **SUPPLEMENTARY RESULTS**

In the main manuscript, occupancy timecourses for a limited set of TSNs were shown. In Figures S2 and S3, we append these primary results by showing trial averaged occupancy timecourses for all 8 TSNs derived using self-paced (figure S2) and Sternberg (Figure S3) datasets. In addition, the Figures also show the mean fractional occupancy (B) (over all time) for each TSN, and the mean canonical correlation (strength of connectivity between clusters) (C) associated with each TSN.

**
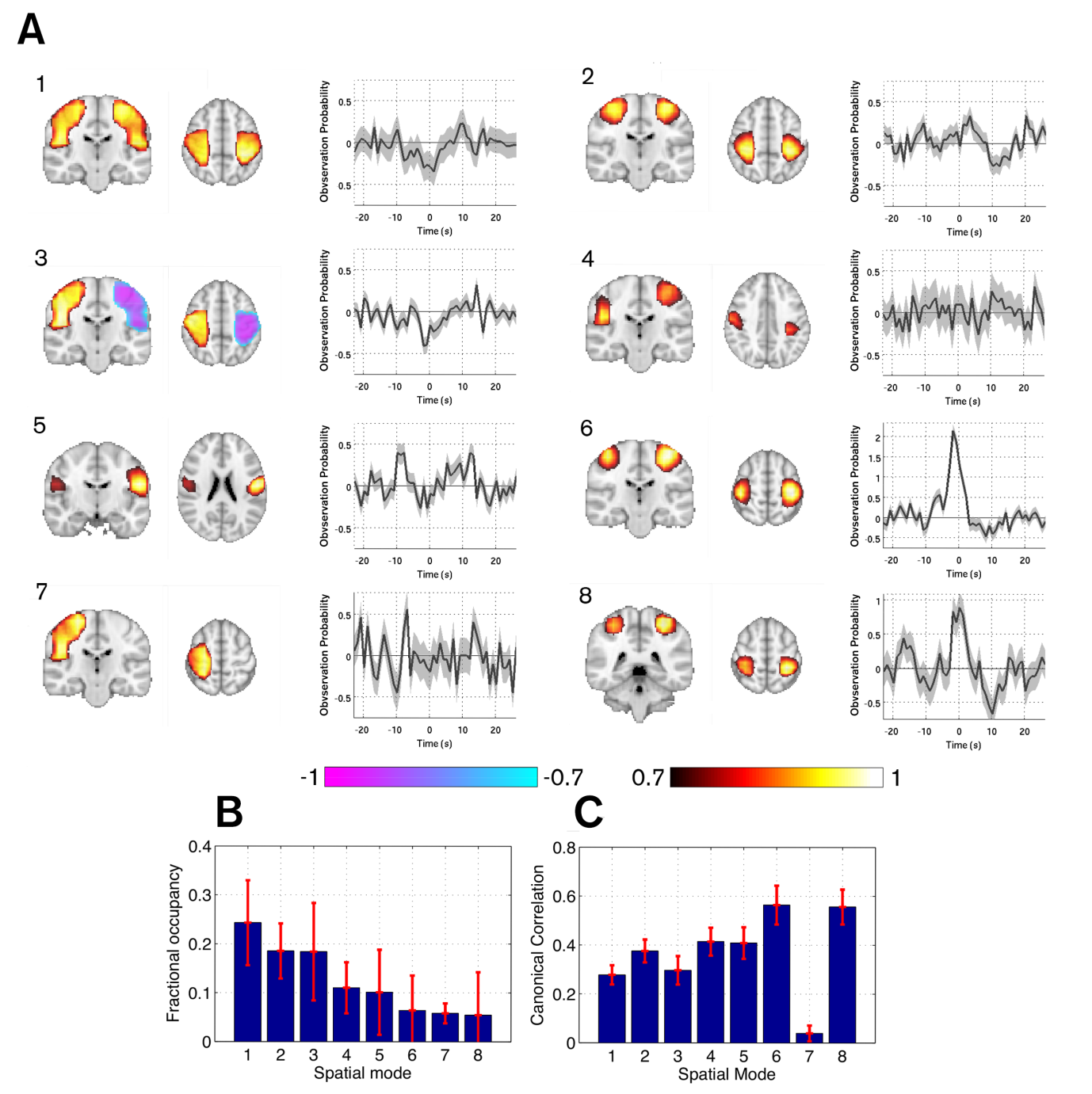
**

***Figure S2:*** *Extended self-paced motor task results. A) Transiently synchronising sub-network (TSN) maps and associated probability timecourses. B) Fractional occupancy (defined as the overall likelihood of seeing each TSN) for the 8 spatial maps. C) Mean canonical correlation coefficient (strength of functional connectivity) associated with each TSN. In B and C, error bars show standard error across subjects.*

**
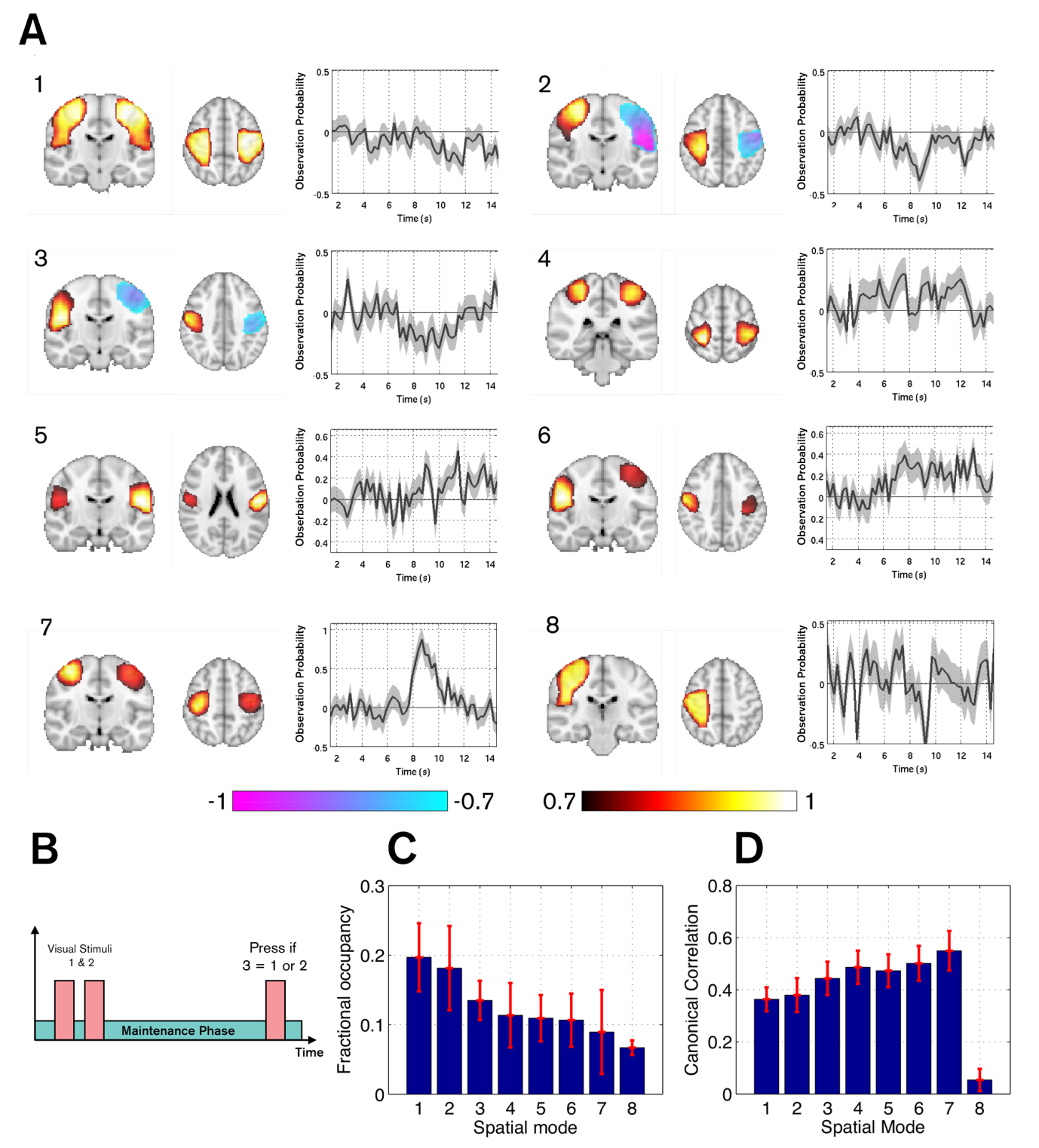
**

***Figure S3:*** *Extended Sternberg task results. A) TSN maps and associated probability timecourses. B) Simple schematic of experiment. C) Mean fractional occupancy for each TSN. D) Mean canonical correlation coefficient for each TSN. In C and D error bars represent error across subjects.*

In the discussion to our main manuscript, we noted that “pure resting state” MEG data are typically recorded when a subject is asked to lie in a system and “think of nothing” whilst imaging data are recorded. Distinct from this, we employed a mixed approach, of interleaving a task with the resting state. This allowed us to both probe the existence of TSNs in the resting state, and validate our methodology with respect to its capability to elucidate temporal fluctuations of sub-network occupancies during the task. However, further validation to test for the presence of TSNs in pure resting state data would be of some value. With this in mind, we applied our technique to a separate multi-subject “pure” resting state dataset.

Ten subjects took part in a ‘pure resting state’ study. Each subject was asked to lie in the scanner with their eyes open and think of nothing whilst 600s of resting state data were acquired using the third order synthetic gradiometer configuration of a 275 channel system, at a sample rate of 600Hz. Data analyses were the same as those used for the self-paced and Sternberg datasets. The spatial signatures of the 8 derived transiently synchronising sub-networks are shown in Figure S4. Note the similarity between what is shown here and the equivalent maps shown in Figures 2, S2 and S3 and that M1 and S1 networks are separated.

**
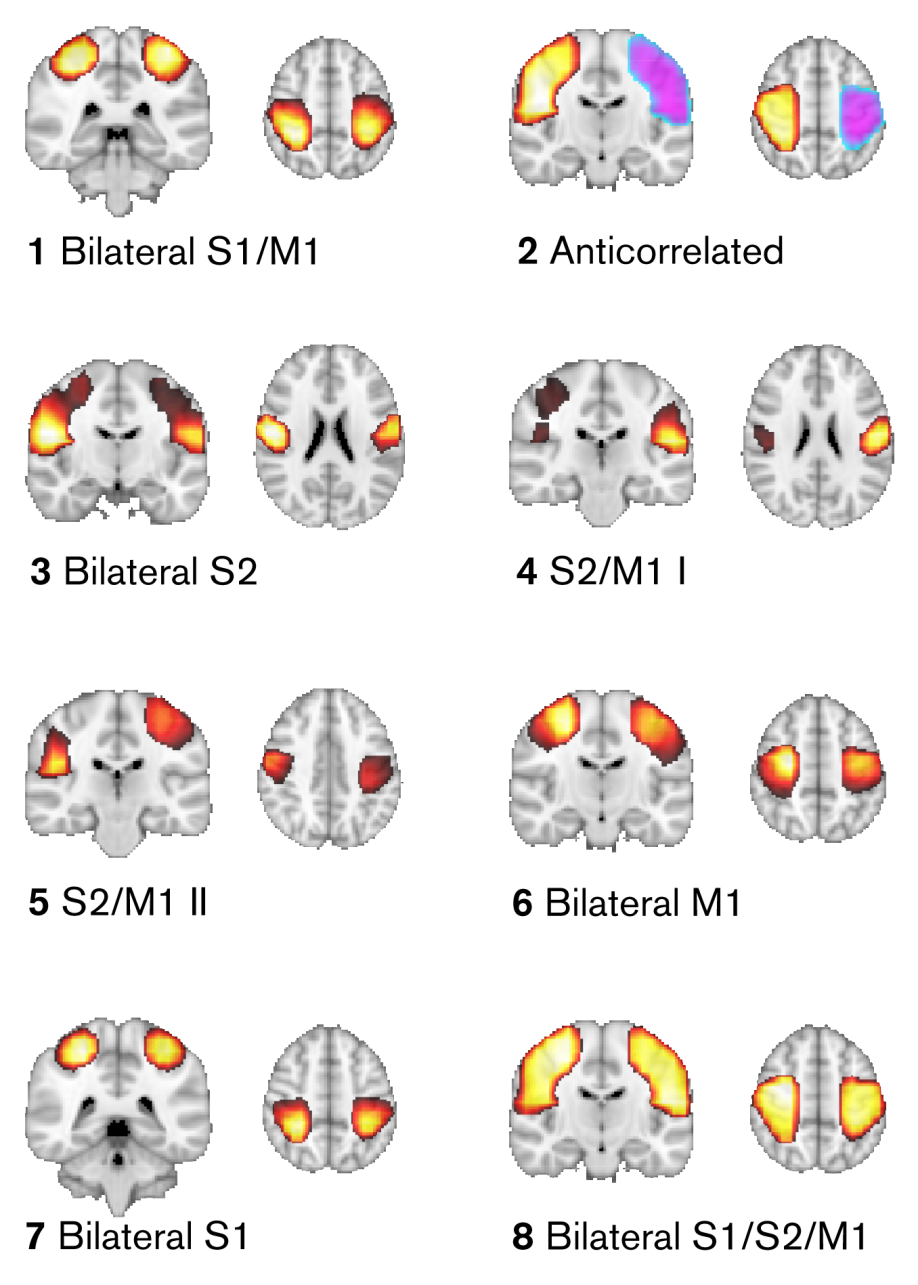
**

***Figure S4:*** *TSN maps derived from pure resting state data in 10 subjects.*

1. **DYNAMIC SIGNAL LEAKAGE REDUCTION**

The major confound of MEG connectivity measurements is signal leakage between source space timecourses ([Brookes et al., 2014](#_ENREF_1); [Brookes et al., 2012](#_ENREF_2); [Hipp et al., 2012](#_ENREF_3); [Maldjian et al., 2014](#_ENREF_4); [Nolte et al., 2004](#_ENREF_5); [Stam et al., 2007](#_ENREF_7)). This is a consequence of the ill-posed MEG inverse problem and the term ‘leakage’ describes the combined effect of the spatial spread of sources (characterised by a point spread function and typically due to correlation between beamformer weights in sensor space) and spatial mis-localisation of sources (for example due to an inaccurate lead field model). Both effects allow true signal from the seed location to ‘leak’ into the estimated signal from the test location (and vice versa). If not taken properly into account, this results in artifactually increased functional connectivity estimates ([Brookes et al., 2012](#_ENREF_2); [Hipp et al., 2012](#_ENREF_3)). It therefore follows that any studies of the type described here must account for this significant confound. Below, we explain, in detail, the methodology used and specifically we show that for non-stationary functional connectivity measurement, if changes in variance of either a seed or test timecourse are expected between windows, then dynamic leakage reduction is essential to ensure unbiased functional connectivity estimaion.

***3.1) Analytical analysis***

It is well known that leakage gives rise to a zero-phase-lag linear interaction between projected signals and this has been exploited in previous methods ([Brookes et al., 2012](#_ENREF_2); [Hipp et al., 2012](#_ENREF_3)) where a post-hoc analysis is used to reduce zero-phase-lag interaction prior to connectivity assessment. Briefly, if ${\hat{\mathbf{q}}}_{\boldsymbol{1}}$ and ${\hat{\mathbf{q}}}_{\boldsymbol{2}}$ represent beamformer projected timecourses from brain locations 1 and 2 (the test and seed locations) respectively, then linear leakage between regions can be reduced via regression of ${\hat{\mathbf{q}}}_{\mathbf{2}}$ (the seed) from ${\hat{\mathbf{q}}}_{\mathbf{1}}$ (the test). The modified test timecourse, ${\hat{\mathbf{q}}}_{\boldsymbol{1}\boldsymbol{M}}$, becomes:

${\hat{\boldsymbol{q}}}_{\boldsymbol{1}\boldsymbol{M}}={\hat{\boldsymbol{q}}}_{\boldsymbol{1}}-{\hat{\boldsymbol{q}}}_{\boldsymbol{2}}\left\{ {\hat{\boldsymbol{q}}}_{\boldsymbol{2}}^{+}{\hat{\boldsymbol{q}}}_{\boldsymbol{1}} \right\}$ [9]

In doing this, we orthogonalise the seed and test timecourse (i.e. ${\hat{\mathbf{q}}}_{\mathbf{1M}}\mathbf{.}{\hat{\mathbf{q}}}_{\mathbf{2}}=0$) and therefore reduce linear leakage. Following orthogonalisation, a non-linear transform is applied to ${\hat{\mathbf{q}}}_{\boldsymbol{1}\boldsymbol{M}}$ and ${\hat{\mathbf{q}}}_{\boldsymbol{2}}$ (computation of the amplitude envelope) and connectivity is calculated. This methodology has the advantage that, since all leakage is zero phase lag, then (given Gaussian assumptions) the effect of leakage must necessarily be reduced in the corrected timecourses prior to connectivity estimation. Furthermore, the magnitude of the leakage between locations 1 and 2 (prior to orthogonalisation) can also be estimated as ${\beta_{L}\mathbf{=}\hat{\mathbf{q}}}_{\mathbf{2}}^{+}{\hat{\mathbf{q}}}_{\mathbf{1}}$.

Unfortunately, there are limitations of this approach. Importantly, the regression method does not make the modified test timecourse, ${\hat{\mathbf{q}}}_{\mathbf{1M}}$, a faithful reconstruction of the true source timecourse. Consider a simple two source model where $\mathbf{q}_{\boldsymbol{1}}$ and $\mathbf{q}_{\boldsymbol{2}}$ represent the genuine electrophysiological timecourses at locations 1 and 2. These sources are estimated via beamformer projection as ${\hat{\mathbf{q}}}_{\boldsymbol{1}}$ and ${\hat{\mathbf{q}}}_{\boldsymbol{2}}$ (i.e. the hat notation represents a beamformer estimate). If we assume that $\mathbf{q}_{\boldsymbol{1}}$ and $\mathbf{q}_{\boldsymbol{2}}$ are zero mean normally distributed processes with variances $\upsilon_{1}^{2}$ and $\upsilon_{2}^{2}$ (see Figure S5) then it is possible to show (see Appendix) that:

[10]

and

[11]

Where *b* represents the magnitude of source leakage from **q_1_** (the test) into **q_2_** (the seed), *k* is a positive constant and *N* represents the number of time points in **q_1_** and **q_2_**. These two Equations reflect the key point that the modified timecourse ${\hat{\mathbf{q}}}_{\mathbf{1M}}$ retains an element from **q_2_** (see Appendix for details). The regression only affects the magnitude of that leakage in such a way as to ensure orthogonality. In addition to this, the regression technique necessarily assumes Gaussian distributed source timecourses (see the appendix of ([Brookes et al., 2014](#_ENREF_1))), and also reduction of leakage comes at the expense of removing any genuine zero phase lag interactions that may exist.

**
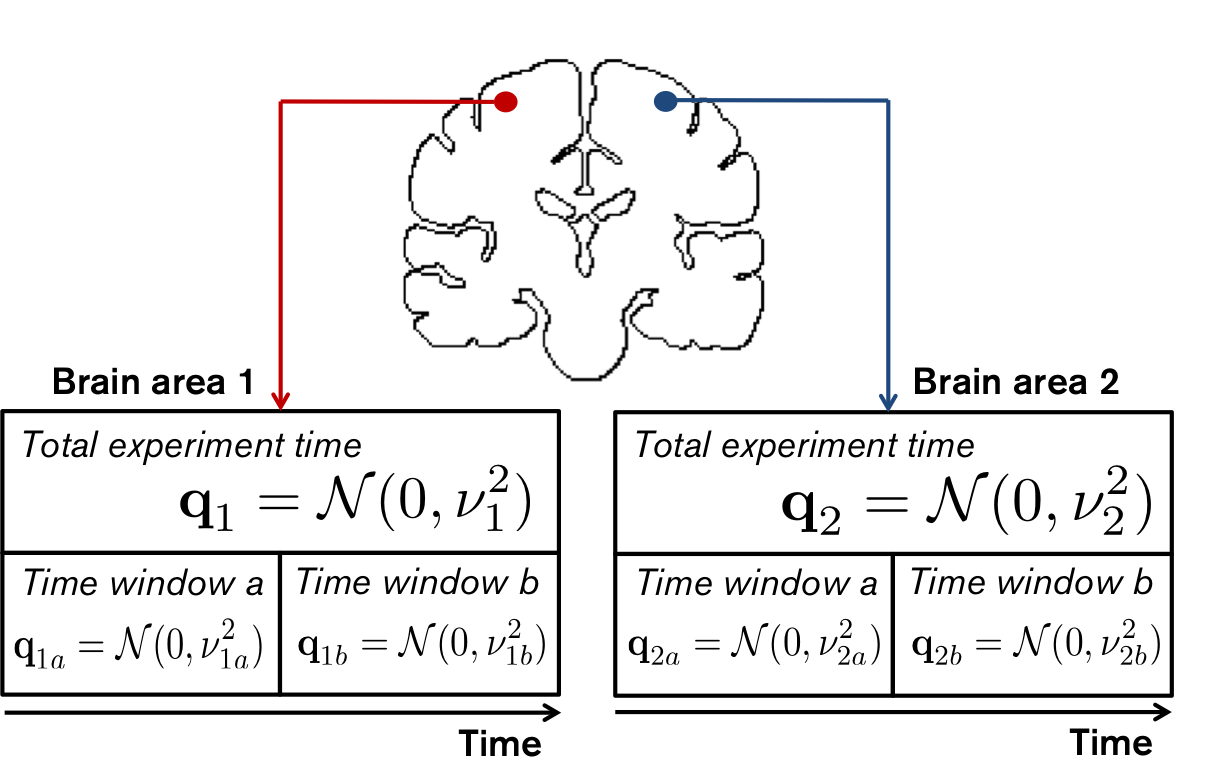
**

***Figure S5:*** *Schematic diagram showing a two source simple model of leakage reduction.*

In addition to the limitations described above, it is unclear how leakage reduction via regression should be deployed in the case of measuring transient and/or task induced functional connectivity. Previous work (based upon resting state data) has taken separate approaches; In some cases ([Brookes et al., 2012](#_ENREF_2)) orthogonalisation is applied over all time, whilst in other cases ([Hipp et al., 2012](#_ENREF_3)) orthogonalisation is undertaken within short time windows. The advantage of the former is that leakage correction will be more precise as it is based on more data. The advantage of the latter is that it will likely be more robust for non-stationary data. However there has, to date, been no systematic investigation into which is most appropriate for measuring transient functional connectivity. Here again our two-source model is of significant utility. Consider the simplest possible case where we split the data into two separate time windows, labelled *a* and *b* (see Figure S5). We further assume that leakage reduction is applied over all time.

It is possible to show analytically (see Appendix) that the magnitude of leakage in time window a, denoted as *r_a_*, is given by:

[12]

Where $\upsilon_{1a}^{2}$ and $\upsilon_{2a}^{2}$ represent the variances of timecourses $\mathbf{q}_{\boldsymbol{1}}$ and $\mathbf{q}_{\boldsymbol{2}}$ in time window *a*. If source variance does not change over time, then $\upsilon_{1a}^{2}=\upsilon_{1}^{2}$ and similarly $\upsilon_{2a}^{2}=\upsilon_{2}^{2}$, meaning that the leakage, *r_a_,* will reduce to zero and therefore leakage reduction over all time represents an adequate strategy. This may be the case when using resting state data with appropriately large time windows where source variance is unlikely to change systematically with the defined windows. However, when measuring task induced transient change, where the window width is purposely made small in order to capture different task phases, variance is highly likely to change across windows (with the task) and so static correction will be inadequate. For example, consider a case where the test source has static variance ($\upsilon_{1a}^{2}=\upsilon_{1}^{2}$) but the seed source has temporal structure; in the case where $\upsilon_{2a}^{2}>\upsilon_{2}^{2}$ (i.e. the variance in window a is larger than the average across all time) static correction would over-correct in time window a, such that . Similarly if $\upsilon_{2a}^{2}<\upsilon_{2}^{2}$, then . This point shows that *if changes in source variance are expected between windows, a dynamic leakage reduction must be performed.*

***3.2) Methods***

*Simulation*

In order to confirm the above analysis and assess the utility of dynamic leakage reduction we undertook a simulation. Six dipoles were simulated in locations of interest within the left and right sensorimotor strips. The first five dipole timecourses comprised 100s of Gaussian distributed data (mean amplitude 1.29 nAm). The sixth dipole timecourse was also Gaussian data but modulated temporally using 5 Hanning windows, each 20s in duration – the standard deviation over all time of this source was 0.8 nAm. These simulated data were projected through a forward model and mixed with empty room noise. We applied source space reconstruction via Beamforming ([Robinson and Vrba, 1999](#_ENREF_6)) to these simulated data and the magnitude of leakage was estimated between voxel clusters covering the left and right sensorimotor strips. Leakage reduction was achieved using a multivariate extension of the regression method which can be found in Brookes et al 2014. Note all six simulated timecourses are uncorrelated and so in the absence of leakage, we would expect to find zero correlation between the left and right clusters. Leakage was assessed in three cases: 1) with no leakage reduction 2) with static leakage reduction and 3) with dynamic leakage applied reduction.

*Experimental Data*

In addition to the simulated case, we also estimated the effect of non-stationary leakage in real data. Leakage between the left and right sensorimotor strips was assessed in a single subject taking part in the self-paced motor task. The sensorimotor strips of the subject’s brain were isolated and masked. Source space data within these masks were reconstructed using the beamformer. Again leakage was reduced using the multi-variate method ([Brookes et al., 2014](#_ENREF_1)) on the reconstructed signals. We then assessed the magnitude of signal leakage with 1) no leakage reduction 2) static leakage reduction and 3) dynamic leakage reduction. The spatial profile of leakage across the left sensorimotor strip was also assessed.

**3.3) Results and discussion**

Figure S6A shows results of the leakage reduction simulation. Figure S6Ai shows the location of the six simulated sources along the left and right sensorimotor strips. Simulated timecourses for each source are also shown (inset) with 5 of the 6 sources having constant variance and the 6th having variance with temporal structure. The leakage profile was calculated between volumes of interest shown by the red overlay and covering the left and right sensorimotor regions. Leakage profile results (which were calculated as the average Pearson correlation between the seed timecourse and the test cluster), are shown in Figure S6Aii: the red timecourse represents leakage with no reduction applied; the green timecourse shows leakage when a static reduction scheme is applied; the blue timecourse shows the case for dynamic leakage reduction. Note first that the leakage estimate contains significant temporal structure. This is most apparent in the case of no leakage reduction where the source timecourse shows clearly that the leakage profile tracks the variance of the modulating source in the seed cluster. It follows that without any leakage reduction applied, the result would not only be artefactually high functional connectivity estimates, but also artefactual functional connectivity estimates with temporal structure. When using static leakage reduction, the effect is reduced but nevertheless the leakage estimate is not driven to zero. When using the dynamic reduction scheme, the leakage estimate is zero, as required.

**
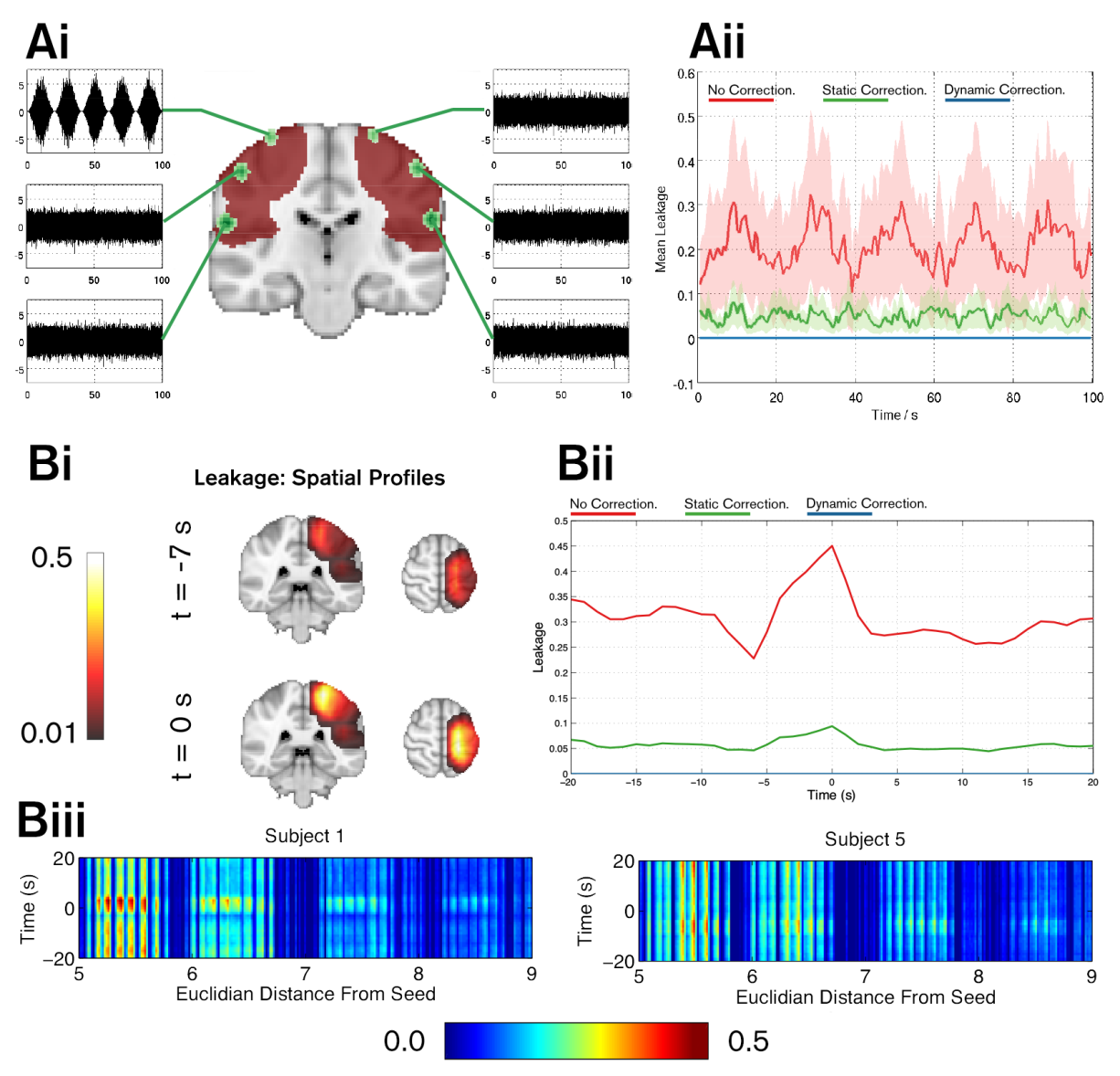
**

***Figure S6:*** *The need for dynamic leakage reduction. A) Results of a 6 source simulation. Ai) The location of the 6 sources (green overlay) alongside the location of the seed and test clusters (red overlay) and the timecourses of each of the simulated sources (inset). Aii) Estimated source leakage from the seed (right sensorimotor strip) to the test (left sensorimotor strip) clusters. No leakage reduction (red), static leakage reduction (green) and dynamic leakage reduction (blue) are shown. B) Leakage in real data. Bi) the spatial profile of leakage for a window not containing a button press (upper panel) and a window containing a button press (lower panel). Bii) Timecourse of leakage estimate for a single voxel, averaged over trials, where 0s corresponds to the button press. Red shows no leakage reduction whilst black shows static leakage reduction – note increased leakage around the time of the event in both cases. Biii) Leakage timecourse for all voxels in the test cluster, for two subjects. Voxels, plotted down the x-axis, are ordered in terms of their Euclidean distance from the seed cluster.*

Figure S6B shows dynamic leakage estimates in real MEG data. Figure S6Bi shows the spatial profile of leakage from the left sensorimotor strip (the seed cluster), into the right sensorimotor strip (the test cluster). The coloured overlay shows the magnitude of the leakage across all voxels in the test cluster. The upper images show the case for a time window not capturing a button press. The lower image shows the case for a time window centred on a button press (in both cases no leakage reduction has been applied). Note that, in support of both our theoretical analyses and the simulation in Figure S6A, temporal structure is observed in the leakage profile in real data. This observation is supported by Figure S6Bii which shows the timecourse of leakage for a single voxel, averaged over all task trials in the self-paced button press paradigm. The red trace shows no leakage reduction whilst the black trace shows static leakage reduction. It is important to note that, even with static leakage reduction, the change in variance of the beta band response, induced in the beamformer projected signal by the task, generates a change in the leakage profile. This in turn could be misinterpreted as a genuine task induced change in functional connectivity but is only a result of the imperfect reconstruction. It is however important to note that this effect is not observed in all subjects; this would be expected since the spatial resolution of the beamformer spatial filter (and therefore the spatial profile of signal leakage) will depend on a number of factors including overall signal to noise ratio of the data and source orientation. This inconsistency is highlighted if Figure S6Biii, which shows the leakage timecourse for all voxels in the test cluster, plotted as a function of Euclidean distance from the centre of the seed cluster, for two subjects. Note that, for subject 1, leakage is task related with a clear increase around the button press (time zero). However, for subject five, no such effect is observed.

**APPENDIX:**

To demonstrate the requirement for dynamic leakage reduction in transient task based MEG connectivity analyses, we assume two sources: **q**_1_ represents the timecourse from a test location (**r**_1_) and **q**_2_ represents the timecourse from a seed location (**r**_2_). The MEG data are described by:

[A1]

Where and represent the lead fields for sources **q**_1_ and **q**_2_ respectively; **e** represents sensor noise. Assuming a beamformer reconstruction, the estimate of **q**_1_ is:

[A2]

Note that represents leakage from the seed, **q**_2_, to the test **q**_1_. Similarly, the beamformer estimate at the seed source **q**_2_ will be:

[A3]

where represents the leakage from the test source. Using the leakage reduction algorithm described above, the leakage estimate, *β*, is given using a regression of the seed voxel onto the test voxel. In the static leakage reduction case, *β* is given by a Moore Penrose pseudo-inverse thus:

[A4]

If **q**_1_ and **q**_2_ are temporally uncorrelated such that , then:

[A5]

where we have used and likewise . Here *N* is the number of samples in the time courses; represents the average variance of source 1 over the entire experiment and similarly represents the average variance of source 2. Having found *β*, it becomes possible to derive an equation for the modified estimated source timecourse () following leakage reduction:

[A6]

Which simplifies to:

[A7]

Where is a constant. This shows that leakage reduction applied in this way does not mean that is a corrected and hence faithful reconstruction of . Rather, it ensures and are orthogonal.

We now use this model to compute what happens in the case of dynamic connectivity estimation. Assume first that the timecourses are broken into windows; for simplicity we employ two contiguous windows, labelled *a* and *b*, such that and . We consider the case where leakage reduction is applied based upon the whole timecourse, as per Equation A6, and we estimate leakage within window a. The leakage estimate for this single window can be computed as the correlation coefficient between and , and should be zero. Mathematically, the correlation coefficient is:

[A8]

Taking , and assuming (assuming that the beamformer covariance is computed over the whole experiment, so *b* is invariant over time) then:

Assuming that the underlying source timecourses in window a are orthogonal ():

[A9]

We can let and ; where and are the variances for sources 1 and 2 in time window a respectively. The N/2 terms reflect the fact that time windows a and b are of length, N/2 samples. Simplifying Equation A9 we arrive at:

[A10]

If the variance of the test source is constant over all time, such that then . We therefore see that only in the case where will the windowed leakage estimate (post static leakage reduction) collapse to the required value of zero. In other words, in cases where the variance of the seed timecourse is invariant across separate windows, a static reduction adequately ensures zero leakage following leakage reduction. However, in cases where the seed variance changes between windows, the estimated leakage is non-zero and leakage reduction is required within each window of interest. Similar arguments can be put forward in the case of varying test signal variance across windows, or in cases where both the seed and test variance change across windows.

**REFERENCES:**

Brookes, M.J., O'Neill, G.C., Hall, E.L., Woolrich, M.W., Baker, A., Palazzo Corner, S., Robson, S.E., Morris, P.G., Barnes, G.R., 2014. Measuring temporal, spectral and spatial changes in electrophysiological brain network connectivity. Neuroimage 91, 282-299.

Brookes, M.J., Woolrich, M.W., Barnes, G.R., 2012. Measuring functional connectivity in MEG: a multivariate approach insensitive to linear source leakage. Neuroimage 63, 910-920.

Hipp, J.F., Hawellek, D.J., Corbetta, M., Siegel, M., Engel, A.K., 2012. Large-scale cortical correlation structure of spontaneous oscillatory activity. Nat Neurosci 15, 884-890.

Maldjian, J.A., Davenport, E.M., Whitlow, C.T., 2014. Graph theoretical analysis of resting-state MEG data: Identifying interhemispheric connectivity and the default mode. Neuroimage 96, 88-94.

Nolte, G., Bai, O., Wheaton, L., Mari, Z., Vorbach, S., Hallett, M., 2004. Identifying true brain interaction from EEG data using the imaginary part of coherency. Clin Neurophysiol 115, 2292-2307.

Robinson, S., Vrba, J., 1999. Functional neuroimaginf by synthetic aperture magnetometry (SAM). In: Yoshimoto, T., Kotani, M., Kuriki, S., Karibe, H., Nakasato, N. (Eds.), Recent advances in biomagnetism. Tohoku University Press, Sendai, pp. 302-305.

Stam, C.J., Nolte, G., Daffertshofer, A., 2007. Phase lag index: assessment of functional connectivity from multi channel EEG and MEG with diminished bias from common sources. Hum Brain Mapp 28, 1178-1193.
